# Effects of *Yucca schidigera* Extract Inclusion in Holstein Calves’ Diets on Performance, Metabolism, and Rumen Volatile Fatty Acid Profile

**DOI:** 10.3390/ani15040566

**Published:** 2025-02-15

**Authors:** Tainara L. dos Santos, Emeline P. Mello, Maksuel G. de Vitt, Michel G. Triantafyllou, Luiz Eduardo Lobo e Silva, Roger Wagner, Aleksandro S. Da Silva

**Affiliations:** 1 Programa de Pós-Graduação em Zootecnia, Universidade do Estado de Santa Catarina (UDESC), Chapecó 89815-630, Brazil; tainaraleticia915@gmail.com (T.L.d.S.); emeline@unochapeco.edu.br (E.P.M.); mak-witt@hotmail.com (M.G.d.V.); 2 Departamento de Zootecnia, Universidade do Estado de Santa Catarina (UDESC), Chapecó 89815-630, Brazil; 05493055996@edu.udesc.br; 3 Departamento de Ciências de Alimentos, Universidade Federal de Santa Maria, Santa Maria 97105-900, Brazil; eduardo.lobo@acad.ufsm.br (L.E.L.e.S.); rogerwag@gmail.com (R.W.)

**Keywords:** additive, acetate, weight gain, growth performance

## Abstract

In the diet of calves, the search for additives that aid in their development is essential due to the impact that these can generate throughout their lives, resulting in a relationship with their longevity and the maintenance of the herd. *Yuca schidigera* has been used in ruminant diets, impacting health and performance; however, there are few reports on its intake by dairy cattle in the rearing phase. Therefore, this study’s objective was to evaluate whether adding *Yucca schidigera* to the diet of calves in the rearing phase can benefit health, productive performance, and feed efficiency. The study was conducted over 60 days, with 24 calves divided into two groups: G1 had no added *Yucca schidigera* and G2 had 188 mg of *Yucca schidigera* added per kg of dry matter intake. Data and samples were collected at 30-day intervals. No treatment effect was observed in body weight, weight gain, daily weight gain, or feed intake (*p* > 0.05); however, the calves in G2 showed 26.3% lower feed efficiency than those in G1. Using the concentrate consumption of the calves, the amount of *Yucca schidigera* ingested was calculated, i.e., 555 mg/calf/day. The total leukocyte count was significantly lower in the calves in G2 compared to G1 on the 60th day. No effect from the treatment or treatment × day interaction was observed for the hematologic variables: erythrocytes, hemoglobin, hematocrit, lymphocytes, granulocytes, monocytes, and platelets. The activities of the enzyme’s gamma glutamyl transferase and aspartate aminotransferase were higher in the animals in group G2. There was no effect from the treatment on albumin, globulin, total protein, C-reactive protein, glucose, cholesterol, urea, or amylase. There was a treatment × day interaction for levels of reactive oxygen species, lipid peroxidation, and glutathione S-transferase, which were higher in the blood of calves in group G2 compared to the control. The activity of myeloperoxidase, superoxide dismutase, and catalase did not differ between groups. Tumor necrosis factor and interleukins (IL-1 and IL-6) in the serum of calves that consumed *Yucca schidigera* were higher on day 60 compared to the control. A higher concentration of short-chain fatty acids in the ruminal fluid was observed in animals in group G2 compared to G1, primarily due to the higher levels of acetic acid. The amounts of propionate and butyrate were lower in the ruminal fluid of calves in group G2. These results allow us to conclude that the consumption of *Yucca schidigera* at a dose of 555 mg/animal/day interferes with calves’ metabolism and negatively affects feed efficiency.

## Simple Summary

*Yuca schidigera Roezl ex Ortgies (Yucca schidigera)* extract at 188 mg/kg DM ingested daily by calves can modulate both the production and the proportion of short-chain fatty acids, increasing acetate and reducing propionate and butyrate. Calves that consumed *Yucca schidigera* extract over an extended period of time showed an increase in inflammatory response biomarkers and elevated oxidative stress, as well as an elevation of liver enzymes that are indications of liver disorders and/or injury. Calves that consumed *Yucca schidigera* had lower feed efficiency.

## 1. Introduction

Milk production in Brazil grew by around 36% from 2018 to 2022 [1]. With the continuous increase in milk production, seeking maximum efficiency throughout an animal’s life cycle is essential. Research on the nutrition of young cattle has become important due to its economic impact related to the performance and health of these animals [2]. Feed additives have shown a significant impact on calf performance [3]. Among them, the use of growth promoters has attracted the attention of the World Health Organization. Since 2006, many countries in the European Union have been banned from using some of these products. Therefore, exploring safe and effective alternatives to replace growth promoters is urgent, with *Yuca schidigera Roezl ex Ortgies* (*Yucca schidigera*) being a natural option that benefits animal performance [4].

Among the various additives, the newest with a nutritional focus is *Yucca schidigera*, which contains secondary bioactives known as steroidal saponins, resveratrol, and other polyphenols [5]. These components, when ingested by animals, can optimize ruminal fermentation, inhibit the action of cellulolytic bacteria and fungi, and, as a result, contribute to the reduction of methane gas (CH^4^) emissions [6]. Using *Yucca schidigera* extract has also positively affected broilers, improving productive performance, carcass characteristics [7], and nutrient digestibility [8]. In dog and cat food, *Yucca schidigera* is used mainly to reduce fecal odor [9].

In cows fed *Yucca schidigera*, there was a reduction in the relative abundance of methanogenic bacteria such as *Methanobrevibacter olleyae*, and these animals had lower feed intake [10]. Research indicates that yucca in the diet of ruminants leads to a significant reduction in CH^4^ concentration [11,12], as well as reducing the emission of nitrous oxide (NH_3_) in feces [13]. In lambs fed *Yucca schidigera*, a lower serum concentration of reactive oxygen species (ROS) and an increase in catalase (CAT) activity were observed, as well as a higher concentration of total protein, triglycerides, and serum globulin [4]. When consuming *Yucca schidigera* extract, Buffalo calves had better growth performance combined with a reduction in ammonia and CH⁴ concentrations [3]. It has been reported that the use of *Yucca schidigera* extract increased levels of immunoglobulins A and G and CAT activity [14]. Therefore, the present study aims to evaluate whether adding *Yucca schidigera* extract to the diet of growing calves positively affects health and growth performance.

## 2. Materials and Methods

### 2.1. Yucca Schidigera Extract

The additive used in this study is a commercial product (De-Odorase^®^, Alltech Serdán, Mexico); the product is composed of a patented extract of the *Yucca schidigera* plant. *Yucca schidigera* was added to concentrate at 250 mg/kg. This dose used was based on a study conducted by our study group with lambs [4], with the precedent that the concentrate was provided at 2% of body weight in both experiments.

### 2.2. Animals and Experimental Location

This research was carried at the experimental farm of the Universidade do Estado de Santa Catarina (FECEO), located in the municipality of Guatambu-SC (Latitude: 27°8′5″ South, Longitude: 52°47′15″ West). The experiment lasted 60 days and included a herd of 24 female Holstein calves, 100 ± 6.5 days old and weighing approximately 90 ± 3.2 kg. The animals were housed in a shed with 12 stalls (1.5 × 7 m) with automatic drinkers, concrete floors, and wooden partitions separating stalls. The animals were housed in pairs of females with similar body weights.

### 2.3. Experimental Design and Diet

The animals were distributed into two groups: G1 (control) (n = 12) and G2 (*Yucca schidigera*) (n = 12). The same basal diet was used for all animals, differing only by addition of the additive; the calves in G2 consumed 188 mg of *Yucca schidigera* extract per kg of dry matter intake. The animals in both groups received the same management regarding light, water, and air conditioning with fans.

The diet was formulated according to the nutritional requirements of the animals, and the nutritional requirements were considered according to the National Research Council (NRC) [15]. The feeds used to formulate the animals’ diet were corn silage, Tifton 85 hay, and concentrate, supplied twice daily (09:00 h and 15:00 h), with a proportion of 66% forage and 33% concentrate (Table S1). The concentrate formulation was 19.33% fine ground corn, 30% soybean meal, 16.66% soybean hulls, 30% wheat bran, 3.2% mineral core, and 0.8% common salt (NaCl). The chemical composition of the total mixed ration (TMR) is presented in Table 1. The samples were pre-dried in a forced ventilation oven at 54 °C for 72 h and ground to facilitate further analyses. The samples were placed in another forced ventilation oven at 105 °C for 24 h to measure and weigh the dry matter (DM). Crude protein quantification was performed following digestion, distillation, and titration using the Kjeldahl method (Method 2001.11) [16]. To obtain ash, the samples were placed in a muffle furnace at 600 °C for six hours, which extracted all the organic matter from the sample, leaving only the ash, which was weighed to obtain the percentage of ash. The quantification of neutral detergent fiber (NDF) and acid detergent fiber (ADF) was performed according to the literature [16,17,18]. According to the manufacturer’s instructions, an automatic fat and lipid extractor (SER 158/6 Velpscientifica^®^, Usmate Velate, Italy) was used to determine the quantification of the ether extract. Results are shown in Table 1.

Concentrate formulated with ground corn (19.33%), soybean meal (30%), soybean hulls (16.66%), wheat bran (30%), mineral core (3.2%), and common salt (NaCl) (0.8%) was combined with 188 mg of *Yucca schidigera* extract per kg of DM intake in animals in group G2. The mineral supplement had the following guaranteed levels: calcium (min) 147.00 g/kg, calcium (max) 179.00 g/kg, phosphorus (min) 120.00 g/kg, sulfur (min) 80.00 g/kg, cobalt (min) 12.50 mg/kg, copper (min) 1250.00 mg/kg, chromium (min) 30.00 mg/kg, iodine (min) 62.50 mg/kg, manganese (min) 2500.00 mg/kg, selenium (min) 25.00 mg/kg, zinc (min) 5000.00 mg/kg, and fluorine (max) 1200.00 mg/kg.

### 2.4. Performance

The experimental unit for the performance variables was the pen, where there were two calves. Therefore, for performance, there were 2 treatments, with 6 replicates per treatment and 2 calves per replicate. Feed intake was measured by weighing the unconsumed feed in the morning before providing the next feed daily per pen (feeding two animals). The calves were weighed thrice (days 1, 30, and 60) using a digital scale (Digitron^®^, Digi-Tron Indústria de Balanças, Curitiba, PR, Brazil). This information was used to calculate weight gain (final weight-initial weight) and average daily gain (ADG) during the 60 days. Using the daily feed intake and ADG, the feed efficiency was calculated (ADG / Daily feed intake).

### 2.5. Sample Collection

Since these were repeated measurements, the experimental unit for blood and ruminal fluid biomarkers was the animal. Therefore, there were 2 treatments, with 12 repetitions each.

Blood was collected from all animals from the jugular vein on days 1, 30, and 60 of the experiment using needles and vacuolated tubes. The collected material was placed in two tubes, one without an anticoagulant, to obtain the serum used in the biochemical analyses, and the other with an anticoagulant (EDTA), for hematologic analyses. The tubes were sent to the laboratory in refrigerated isothermal boxes at 10 °C. The blood samples in tubes without anticoagulant were centrifuged at 7000 rpm for 10 min to obtain serum for biochemical analysis. The samples were then stored at −20 °C until laboratory analyses were performed.

Ruminal fluid was collected on day 60. For collection, the animals were restrained in a restraint trunk, and using a mouth opener and a silicone esophageal probe coupled to a vacuum system (two-way vacuum pump coupled to a glass collecting flask), a 150 mL sample was collected by suction. The pH of the collected material was measured immediately after collection using a portable pH meter. Then, the ruminal fluid material was filtered, and collected material was placed in microtubes and stored (−20 °C) until analysis.

### 2.6. Hemogram

The blood count was performed using an automatic hematology analyzer (EQUIP VET^®^ 3000, Equip, Itatiba, SP, Brazil) and focused on the erythrocyte count (×10^6^/µL), total leukocyte count (differentiated lymphocytes, granulocytes, and monocytes; ×10^3^/µL), and platelets (×10^3^/µL), as well as the hemoglobin concentration (mg/dL) and hematocrit percentage (%).

### 2.7. Metabolic Biochemistry

For biochemical analyses, semi-automatic Bio Plus equipment (Bio-2000^®^, Bioplus Produtos para Laboratórios Ltda, Barueri, SP, Brazil) was used, and serum levels of albumin, total protein, cholesterol, triglycerides, metabolic alanine aminotransferase (ALT), aspartate transaminase (AST), and urea were evaluated using commercial kits (Analisa^®^, Gold Analisa Diagnóstica Ltda, Belo Horizonte, Brazil). The globulin concentration (total protein-albumin) was calculated.

### 2.8. Status Oxidative

Levels of ROS in the serums were determined by the 2′,7′-dichlorofluorescein diacetate (DCF-DA) oxidation method as described by Ali et al. [19], using excitation and emission of the wavelengths of 485 and 538 nm, respectively. Lipoperoxidation is a highly rapid reaction formed by the breakdown of polyunsaturated fatty acids, which are usually measured by their products, mainly thiobarbituric acid reactive substances (TBARS), among which malondialdehyde (MDA) is the primary one [20]. Myeloperoxidase (MPO) is a heme enzyme produced by inflammatory mediators and released from leukocytes at the injury site; therefore, MPO reflects the activation of neutrophils and lymphocytes. MPO catalyzes the reaction of chloride ions with H_2_O_2_ to generate large amounts of hypochlorous acid (HOCl), an ROS that further reacts to generate singlet oxygen and hydroxyl radicals. In the presence of H_2_O_2_ as an oxidizing agent, MPO catalyzes the oxidative coupling of phenol and 4-aminoantipyrine (AAP), originating a colored product, quinonimine, with a maximum absorbance of 492 nm [21]. The results were expressed as μM of quinonimine per mg of protein produced in 30 min (μMq/mg/30 min).

Catalase (CAT) and superoxide dismutase (SOD) activity were measured in the whole blood samples; glutathione S-transferase (GST) was measured in the serum. GST activity was measured according to Habig et al. [22]. SOD activity measurement was based on the inhibition of O_2_ reaction with adrenaline, as described by McCord and Fridovich [23]. CAT activity was quantified using a modified method by Nelson and Kiesow [24].

### 2.9. Cytokine Levels

Cytokine levels in serum were measured. Interleukin 1β (IL-1β), IL-6, and IL-10 were measured using commercial kits and an enzyme-linked immunosorbent assay device (Awareness Technology, Inc., New York, NY, USA; Chem Well; USCN, Wuhan, China).

### 2.10. pH and Short-Chain Fatty Acids in Ruminal Fluid

Ruminal fluid samples were thawed at 5 °C and manually shaken for homogenization. Aliquots of 1 mL of the supernatant of ruminal fluid samples were collected in polypropylene microtubes (2 mL) and then centrifuged for 5 min (12,300× *g*). Then, 250 μL of the supernatant was transferred to a new microtube containing 250 μL of formic acid. The mixture was manually shaken and centrifuged for 3 min. After centrifugation, 250 μL of the supernatant of the mixture was collected in another polypropylene tube previously containing 500 μL of isoamyl alcohol solution (692.40 μg mL^−1^ in methanol), used as an internal standard, and was homogenized and centrifuged again. Following this, 650 μL of the sample was inserted into a 2 mL injection vial; 1 μL of the extract was injected into a gas chromatograph equipped with a flame ionization detector (GC-FID; Varian Star 3400, Hansen Way, Palo Alto, CA, USA) and an autosampler (Varian 8200CX, Hansen Way Palo Alto, CA, USA) in split mode (1:10) at 250 °C. The carrier gas used was hydrogen at a constant pressure of 10 psi.

The analytes (acetic, propionic, butyric, valeric, and isovaleric acids) were separated by a CP-Wax 52CB capillary column (50 m × 0.32 mm, 0.20 μm stationary phase thickness). The initial column temperature was set at 40 °C for 1 min and increased to 100 °C at a rate of 10 °C min^−1^, then to 110 °C for 3.5 °C min^−1^, and finally, to 230 °C at a rate of 20 °C min-1, where it remained for 1 min. The detector temperature was set to 250 °C. Method validation comprised the following parameters: selectivity, linearity, linear range, repeatability, precision, limit of detection (LOD), and limit of quantification (LOQ) for acetic, propionic, butyric, and isovaleric acids. Linearity was assessed by calculating a regression equation using the least squares method. LOD and LOQ values were obtained using sequential dilutions to signal-to-noise ratios of 3:1 and 6:1, respectively. Precision was assessed by analyzing the repeatability of six replicated samples. Accuracy was determined by recovering known amounts of the standard substances added to a diluted sample (Table S2). Valeric acid was expressed as the equivalent of isovaleric acid. The results were expressed in mol 100 mol^−1^ of each short-chain fatty acid (SCFA) in the ruminal fluid.

### 2.11. Statistical Analysis

The experimental data were first analyzed descriptively; measures of central tendency (median) and data dispersion (range that stands for the interval between the minimum and maximum values in the data) were computed. Furthermore, all variables were subjected to the Shapiro–Wilk test, which revealed a normal data distribution. Skewness, kurtosis, and homogeneity were evaluated using the Levene test, and linearity was assessed using linear regression. All data were analyzed using the ‘MIXED procedure’ of SAS (SAS Inst. Inc., Cary, NC, USA; version 9.4), with Satterthwaite approximation to determine degrees of denominator freedom for the fixed-effects test. Weight gain, ADG, and feed efficiency were tested for treatment-fixed effects using pen (treatment) and animal (pen) as random variables (i.e., pen within treatment and animal within pen). Body weight and feed intake were tested for treatment-fixed effects using a pen (treatment) and animal (pen) as repeated measures and tested for treatment, day, and treatment × day interaction. Ruminal fluid biomarkers were tested for treatment-fixed effects using animal (treatment) as a random effect inside each pen. Complete blood count (CBC), biochemistry, cytokines, and oxidative biomarker data were analyzed as repeated measures and tested for treatment, day, and treatment × day interaction as fixed effects using animal (treatment) as a random effect inside each pen. The d 1 results were included as an independent covariate. Additionally, for these variables, the d 1 results were removed from the data set to generate the mean by treatment but were retained as a covariate. The first-order autoregressive covariance structure was selected according to the lowest Akaike information criterion. Means were separated using the PDIFF (*t*-test) method, and all results were reported as LSMEANS followed by SEM. Significance was defined as when *p* ≤ 0.05 and trend when *p* > 0.05 and ≤0.10.

## 3. Results

### 3.1. Performance

Growth performance results are presented in Table 2. There was no difference between groups in body weight, weight gain, daily weight gain, and feed intake (*p* > 0.05); however, lower feed efficiency (26.3%) was observed in G2 animals compared to G1.

Using the animals’ feed intake, it was calculated and verified that the calves consumed 555 mg daily, corresponding to a concentration of 188 mg/kg of DM ingested daily.

### 3.2. Hematology and Serum Biochemistry

Hematology and biochemistry results are presented in Table 3. There was a treatment x day interaction for total leukocyte count, which was lower in G2 calves on day 60 of the experiment. No difference between groups and treatment × day interaction was observed for the other hematologic variables investigated (erythrocytes, hemoglobin, hematocrit, lymphocytes, granulocytes, monocytes, and platelets). GGT and AST activity was higher in G2 animals compared to G1; there was also a treatment × day interaction for AST, with higher activity in the serum of G2 calves on days 30 and 60. There was no difference between groups for the other biochemical variables: albumin, globulin, total protein, C-reactive protein, glucose, cholesterol, urea, and amylase.

### 3.3. Oxidative Biomarkers

Table 4 presents the results of oxidative status. There was a treatment × day interaction for ROS levels, which were higher in group G2 on day 60. TBARS levels were higher in the serum levels of G2 compared to G1. For GST, activity was higher on days 30 and 60 in serum from group G2. There was no difference between groups for myeloperoxidase, superoxide dismutase, or catalase activity.

### 3.4. Cytokines Concentration

**Figure 1:**
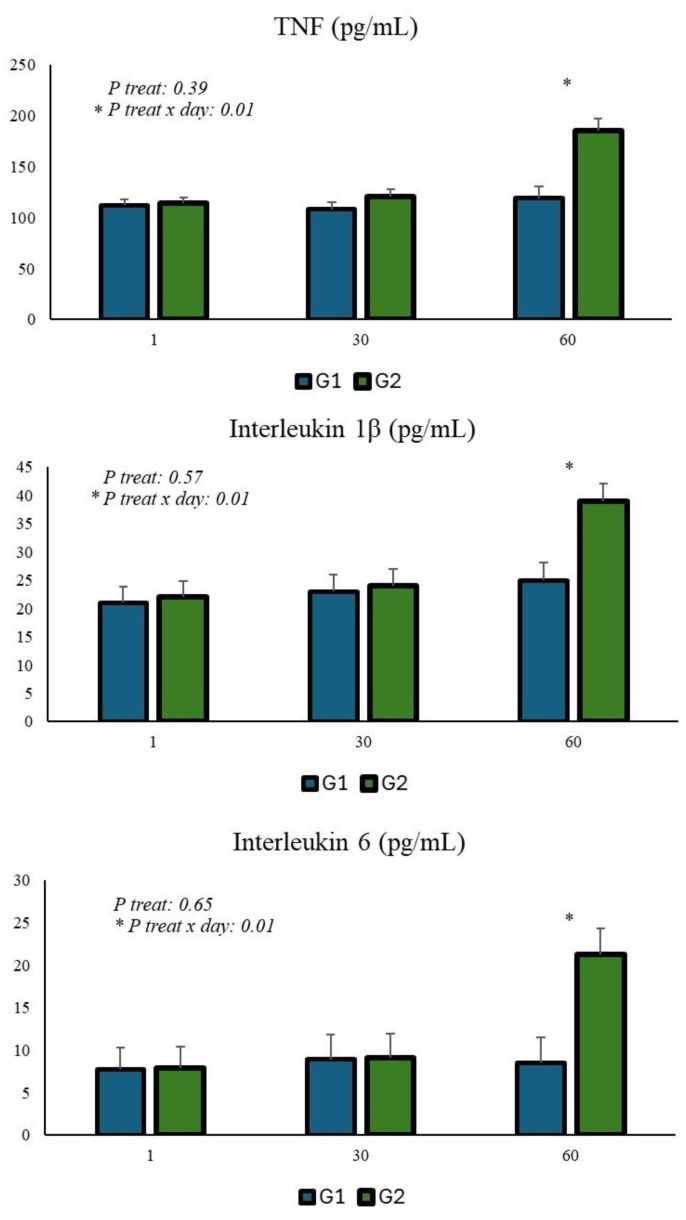
Levels of TNF and interleukins (IL-1 and IL-6) in the serum of calves.

Cytokine results are presented in Figure 1. A treatment x day interaction was used to determine TNF, IL-1, and IL-6. On day 60, higher tumor necrosis factor (TNF), IL-1, and IL-6 serum levels were observed in calves that consumed *Yucca schidigera* extract compared to the control.

### 3.5. Short-Chain Fatty Acids in Ruminal Fluid

Results of ruminal fluid biomarkers are shown in Table 5, with volatile fatty acid (VFA) data presented in two forms: absolute number and percentage. A higher concentration of SCFAs was detected in calves from group G2. In both forms, a higher quantity/proportion of acetic acid was observed in G2, unlike propionic and butyric acid, which was higher in G1. No difference was observed for isovaleric acid or pH in ruminal fluid between groups.

## 4. Discussion

The lower feed efficiency of Holstein calves was not expected, because the results published so far with ruminants show benefits in performance or non-interference, for example, in a study conducted by the same research group [4], and other previously published studies [12,14]. For example, the research by Abdel-Raheem et al. [1] revealed that using *Yucca schidigera* powder improved feed efficiency in buffalo calves, evidenced by more significant nutrient digestibility and increased weight gain. Furthermore, the study by Griss et al. [4] indicated that using *Yucca schidigera* extract in combination with organic chromium in lambs improved feed conversion and enhanced weight gain.

There was a lower leukocyte count in the blood of calves that consumed *Yucca schidigera* extract; no change between groups in the leukocyte differential was observed. Therefore, two hypotheses for this reduction are possible. The first is the anti-inflammatory action of the additive [25] or stress factors caused in the animal by the diet; this type of stress can be caused by metabolic changes associated with the digestion of compounds that induce harmful inflammatory or metabolic responses [26]. The second hypothesis is in line with the higher GGT and AST activity in calves that consumed *Yucca schidigera* extract. According to the literature, these elevated enzymes indicate liver injury or overload [27,28]. The use of high doses or saponins in smaller ruminants can cause adverse effects on animal health due to their complexity of action, which varies according to the species and the dose administered [29].

Free radicals, when at exacerbated levels, cause oxidative damage that affects physiological and metabolic processes, but at normal levels, mainly from cellular respiration, they are essential for the oxidative balance of the organism [30]. In the present study, a 50% increase in ROS levels was observed after 60 days of consumption of *Yucca schidigera* extract, and this increase in ROS may have had deleterious effects on proteins, lipids, or nucleic acids, leading to functional and structural changes in cells, resulting in impairment of cellular function and the general health of the animal [31]. An increase in lipid peroxidation occurred, as there was a 39% increase in TBARS levels, a sensitive signal of lipid cell damage. This increase suggests oxidative reactions are associated with an inflammatory response, since pro-inflammatory cytokines (TNF, IL-1, IL-6) also increased after 60 days of *Yucca schidigera* consumption. The combination of these results shows that the dose of *Yucca schidigera* used caused an inflammatory response and oxidative stress, which explains the lower numerical weight of these animals, which consumed more and made less use of nutrients.

The hypothesis is that the increase in serum ROS and TBARS-associated cytokines may reflect tissue inflammation, most likely in the intestine, which reduced absorption ability, affecting weight gain and worsening feed efficiency. Our research group will explore these speculations in future studies. However, it is important to note here that the ingestion of a combination of probiotics, prebiotics, and essential oils (dose 1.5 kg/t) in post-weaning calves also increased ROS levels, as well as SOD activity, in the animals’ blood [32]. Likewise, there was an increase in GST activity, indicating an antioxidant response to neutralize elevated free radicals, thus protecting cells against oxidative damage, especially in the liver. This protection is essential for maintaining cellular homeostasis and preventing diseases related to oxidative stress [33,34].

A higher concentration of SCFAs was found in Holstein calves that consumed *Yucca schidigera* extract. It is already known that the use of natural additives in feed influences the rumen fermentation process, modulating the absorption of nutrients, which can result in benefits in the weight gain of cattle [35] or in milk quality [36]; however, it is important to make it clear that the dose of these additives is currently the most substantial difficulty, as it changes according to the animal species and according to the production category or diet, as had already been observed by other researchers who studied phytogenetics [37]. However, in the present study, a higher proportion and quantity of SCFAs did not result in productive efficiency or weight gain. Holstein calves that consumed *Yucca schidigera* had a higher concentration of acetic acid and a lower concentration of propionate and butyrate. Most studies involving *Yucca schidigera* tend to report increased propionate production, which occurred in heifers [38] and buffalo calves [3]. In the latter, in addition to the increase in propionate production, a linear reduction in the acetate/propionate ratio was identified as *Yucca schidigera* levels increased, resulting in a decrease in cellulolytic bacterial growth [3]. The influence of *Yucca schidigera* extract on acetic acid production in the rumen is complex and may be multifactorial, conditioned by variables such as the dosage of the additive, the composition of the diet, and the interaction with the ruminal microbiota. Future research can be conducted to determine the optimal dose for calf health and performance.

## 5. Conclusions

*Yucca schidigera* extract at 188 mg/kg DM ingested daily by calves can modulate both the production and the proportion of SCFAs, increasing acetate and reducing propionate and butyrate. Calves that consumed *Yucca schidigera* extract over an extended period of time had an increase in inflammatory response biomarkers and elevated oxidative stress, as well as an elevation of liver enzymes that are indications of liver disorders and/or injury. Consequently, calves that consumed *Yucca schidigera* extract had lower feed efficiency.

## Figures and Tables

**Table 1:** Chemical composition of TMR used in the study.

Variables	TMR: Control	TMR: *Yucca schidigera*
DM, %	46.6	47.6
Ash, %	7.28	7.44
Crude protein, %	12.6	12.7
NDF, %	46.3	45.2
EE, %	3.52	3.85

**Table 2:** Body weight, weight gain, ADG, feed intake, and feed efficiency of calves not receiving (G1) or receiving (G2) *Yucca schidigera* extract in their diets.

Variables	G1	G2	SEM	*p*-Value
Initial weight, kg	95.8	98.2	2.54	0.94
Final weight, kg	135.2	133.1	2.76	0.81
Weight gain, kg	39.4	34.9	1.78	0.16
ADG, kg	0.65	0.58	0.05	0.15
Daily feed intake, kg MS	2.69	2.95	0.11	0.52
Feed efficiency, kg/kg	0.24 ^a^	0.19 ^b^	0.02	0.05

Note: *p* ≤ 0.05 indicates treatment effect, illustrated by different letters on the same line.

**Table 3:** Serum hematologic and biochemical biomarkers in calves not receiving (G1) or receiving (G2) *Yucca schidigera* extract in their diets.

Variables	G1	G2	SEM	*p*-Treat	*p*-Treat × Day
Total leukocytes (×10^3^ µL)				0.36	0.05
d1	7.05	6.85	0.29		
d30	6.11	5.56	0.31		
d60	7.17 ^a^	5.18 ^b^	0.43		
Lymphocyte (×10^3^ µL)	3.71	2.91	0.31	0.31	0.13
Granulocyte (×10^3^ µL)	2.21	1.88	0.27	0.45	0.29
Monocyte (×10^3^ µL)	0.62	0.67	0.04	0.86	0.77
Erythrocytes (×10^6^ µL)	7.29	7.95	0.20	0.88	0.93
Hemoglobin (mg/dL)	9.91	10.1	0.21	0.92	0.95
Hematocrit (%)	27.0	28.2	0.62	0.91	0.86
Platelets (×10^3^ µL)	451	477	17.2	0.73	0.82
Albumin (g/dL)	2.83	2.91	0.04	0.92	0.89
Amilase (U/L)	105	114	9.63	0.81	0.66
Cholesterol (mg/dL)	67,2	63.1	1.81	0.49	0.37
GGT (U/L)	8.59 ^b^	15.7 ^a^	3.41	0.05	0.17
Glucose (mg/dL)	83.5	82.7	2.83	0.92	0.79
C-reactive protein (mg/dL)	3.15	3.15	0.03	0.98	0.97
Total protein (g/dL)	7.08	6.89	0.10	0.74	0.81
Urea (mg/dL)	29.4	29.5	0.77	0.96	0.86
Globulin (g/dL)	4.25	3.97	0.08	0.23	0.12
AST (U/L)				0.02	0.01
d1	91.8	87.6	2.79		
d30	68.5 ^b^	86.4 ^a^	2.57		
d60	69.3 ^b^	80.8 ^a^	2.41		

Note: *p* ≤ 0.05 indicates treatment effect and treatment × day interaction, illustrated by different letters on the same line.

**Table 4:** Serum oxidative status biomarkers in calves not receiving (G1) or receiving (G2) *Yucca schidigera* extract in their diets.

Variaveis	G1	G2	SEM	*p*-Treat	*p*-Treat × Day
ROS (U DCF/mL)				0.21	0.01
d1	752	725	8.25		
d30	658	698	8.96		
d60	642 ^b^	967 ^a^	10.4		
TBARS (nmol MDA/mL)	15.6 ^b^	21.7 ^a^	1.28	0.05	0.11
MPO (uM de quinoneimine/30 min)	5.24	5.36	0.11	0.85	0.91
SOD (U SOD/mg protein)	12.4	15.1	1.05	0.12	0.25
CAT (U CAT/mg protein)	2.71	3.24	0.38	0.18	0.11
GST (U GST/mg of protein)				0.01	0.01
d1	235	242	6.84		
d30	231 ^b^	305 ^a^	7.95		
d60	228 ^b^	297 ^a^	7.55		

Note: When *p* ≤ 0.05, there was a treatment effect and treatment × day interaction, illustrated by different letters on the same line.

**Table 5:** Fatty acid profile of ruminal fluid in calves not receiving (G1) or receiving (G2) *Yucca schidigera* extract in their diets.

Variables	G1	G2	SEM	*p*-Value
Total SCFAs, mmol/L	40.5 ^b^	48.2 ^a^	2.20	0.05
*Levels*				
Acetic acid, mmol/L	30.0 ^b^	39.7 ^a^	1.94	0.03
Propionic acid, mmol/L	5.82 ^a^	4.53 ^b^	0.37	0.09
Butiric acid, mmol/L	4.04	3.49	0.33	0.36
Isovaleric acid, mmol/L	0.70	0.88	0.09	0.59
*Percentage*				
Acetic acid, %	74.0 ^b^	81.5 ^a^	1.20	0.01
Propionic acid, %	14.5 ^a^	9.82 ^b^	0.99	0.01
Butiric acid, %	9.77 ^a^	6.92 ^b^	0.52	0.02
Isovaleric acid, %	1.63	1.74	0.14	0.52
pH	7.13	7.15	0.04	0.94

Note: *p* ≤ 0.05 indicates treatment effect, illustrated by different letters on the same line.

## Data Availability

The original contributions presented in the study are included in the article, further inquiries can be directed to the corresponding author.

## Supplementary Materials

The following supporting information can be downloaded at https://www.mdpi.com/article/10.3390/ani15040566/s1: Table S1. Ingredients and chemical composition of ingredients and experimental feeds; Table S2: Standardization of the quantification analysis of SCFAs in the ruminal fluid of cattle.
