# Supplementary material for: Effects of Yucca schidigera Extract Inclusion in Holstein Calves’ Diets on Performance, Metabolism, and Rumen Volatile Fatty Acid Profile
Source: Animals (Basel). 2025 Feb 15;15(4):566. doi: 10.3390/ani15040566 (PMC11851949; doi:10.3390/ani15040566)
Supplement: Supplementary file 1 [file animals-15-00566-s001.zip › animals-3459179-supplementary.pdf]

Table S1. Ingredients and chemical composition of ingredients and experimental feeds.

Ingredients	Composition TMR (%)[1]			
Corn silage	62.6			
Hay: Tifton 85	3.55			
Concentrate	33.8			
**Calculated composition[2] (%)**	**Corn Silage**	**Hay**	**Concentrate**	**Yucca extract**
DM	33.2	84.2	96.2	100
Starch	28.5	-	-	-
CP	8.59	11.8	17.3	1.88
NDF	44.0	74.2	21.5	1.81
EE	3.54	1.61	1.27	1.10
Ash	5.14	6.90	9.47	2.23

[1] Composition in Body Weight

[2] Dry Matter composition: Dry Matter (DM), Crude Protein (CP), Neutral Detergent Fiber (NDF), Ether Extract (EE), ASH (ash)

The composition of the TMR between groups G1 and G2 differs only in 188 mg of yucca extract per kg of DM intake in animals in group G2

Table S2. Standardization of the quantification analysis of short-chain fatty acids in the ruminal fluid of cattle.

Marked	**Acetic acid**	**Propionic acid**	**Butyric acid**	**Isovaleric acid**
R^2	0.9992	0.9991	0.9994	0.9993
Equation	y = 0.0095x + 0.0158	y = 0.0166x - 0.0073	y = 0.0226x + 0.0016	y = 0.0313x - 0.0009
Linear range (mmol L^{-1})*	4.50 - 135.11	1.67 - 133.24	1.41 - 33.91	0.60 - 14.37
LOD (mmol L^{-1})	1.13	0.83	0.71	0.30
LOQ (mmol L^{-1})	4.50	1.67	1.41	0.60
Accuracy	101.76	104.61	105.63	99.19
Repeatability (RSD)	3.70	4.52	3.97	3.24

* The linear range. LOD (limit of detection) and LOQ (limit of quantitation) were expressed in mmol of SFA for L of ruminal fluid
